# Afebrile chemotherapy-induced neutropenia: an international survey spots oncologists’ routine clinical practice versus the standard of care and the impact of COVID-19

**DOI:** 10.1007/s00520-022-07421-8

**Published:** 2022-10-29

**Authors:** Ereny Samwel Poles Saad, Karima Oualla, Narmin Talibova, Snezhanna Gening, Shady Gayed YousefYousef

**Affiliations:** 1 Faculty of Medicine, Clinical Oncology Department, Assiut University, Assiut Governorate, Kornish Al Ibrahimeya, Asyut Second, 71515 Egypt; 2 Medical Oncology Department, Haasan II University Hospital, Sidi Mohamed Ben Abdellah University, Fes, Morocco; 3 Chemotherapy Department, National Oncology Centre, Baku, Azerbaijan; 4 Ulyanovsk State University, Ulyanovsk, Russia; 5 Faculty of Medicine, Assiut University, Asyut, Egypt

**Keywords:** Chemotherapy-induced neutropenia, Indications for G-CSF in afebrile chemotherapy-induced neutropenia, COVID-19

## Abstract

**Introduction:**

Afebrile chemotherapy-induced neutropenia represents a frequent clinical situation where chemotherapy protocol, patient’s comorbidities, and disease status determine the risk of infection hence the management plan. Internationally distributed, this questionnaire aims to evaluate the routine practice and the impact of the COVID-19 pandemic on afebrile chemotherapy-induced neutropenia management.

**Material and methods:**

Coordinators from Egypt, Morocco, Azerbaijan, and Russia developed a 12-item questionnaire using Google forms to explore how oncologists deal with afebrile chemotherapy-induced neutropenia. The link to the survey was available internationally through social media and to their local societies over the period from July to September 2021.

**Results:**

We received 151 responses from 4 world regions: 58.9, 9.9, 11.3, and 15.2% from the Mena area, Russia, Europe, and Asia. The responses deviated from the guideline-driven practice as G-CSF was the most chosen option for intermediate risk that was statistically different based on the academic background of the treating physician. Half of the responders ignored patients and disease risk factors in the intermediate-risk cases that trend was statistically different based on the geographical distribution. The steroid was a valid option for intermediate and low-risk as per oncologists practicing in Russia. COVID-19 pandemic positively affected the rate of prescription of G-CSF as expected.

**Conclusion:**

The disparities in the routine practice of oncologists based on their geographical and academic backgrounds highlight the need to analyze the underlying obstacles that hinder guideline-based practice like workload or lack of the proper knowledge.

## Introduction

Chemotherapy-induced neutropenia is a common chemotherapy-related side effect; it is evident at the lab level with no specific symptoms in majority of cases, while the red flag is fever [1]. Neutropenia increases the risk of morbidity and mortality, and it affects treatment efficacy by delays and dose reduction of subsequent chemotherapy cycles [2]. The grades of neutropenia include mild with absolute neutrophil count (ANC) that is less than 1500 cells/mm^3^, moderate with ANC less than 1000 cells/mm^3^, and severe neutropenia with ANC less than 500 cells/mm^3^ [3]. Febrile neutropenia, when the patient has neutropenia with an oral temperature of ≥ 38.0 °C, results in high morbidity and mortality rates [4]. Factors that raise the risk of febrile neutropenia include chemotherapy protocols, patient comorbidities, and disease stage. The EORTC, ASCO, and the NCCN guidelines have identified the neutropenia risk for different protocols of conventional and dose dense regimens [5–7]. The chemotherapy regimens triage cancer patients to either the high-risk group (> 20% risk of FN), intermediate-risk group (10–20% risk), or the low-risk group (< 10% risk) [8, 5, 6]. Advanced age (> 65) years old and comorbidities like liver dysfunction and cardiac problems are the most common patients’ related risk factors, while the advanced stage of cancer and hematological malignancies are the disease risk factors of concern [9, 10]. The granulocyte colony-stimulating factor (GCSF) is the cornerstone of the primary and secondary prophylaxis of chemotherapy-induced-neutropenia as it reduces the risk of mortality and infection [11]. The prophylactic use of GCSF has a cost-effective value by reducing the frequency of hospitalization due to repeated infection [11, 12]. Studies showed deviations from guidelines in the management of febrile [13] and afebrile neutropenia [14, 15]. The current situation of COVID-19 pandemic tops up the uncertainties about the indication of the GCSF in patients receiving chemotherapy [16].

This questionnaire represents an international collaboration of oncologists practicing in Egypt, Morocco, Azerbaijan, and Russia to evaluate the current practice of the use of G-CSF at the international level and the impact of the current pandemic on it.

## Materials and methods

### Selection of the participants

The questionnaire was available to oncologists (medical, clinical, radiation, surgical) worldwide. Participants were invited to complete a web-based survey to self-evaluate their knowledge, attitude, and behavior regarding the use of G-CSF in daily practice with special concern about changing the routine practice as a result of the recent COVID-19 pandemic.

### Survey distribution and data collection

A 12-item questionnaire was prepared by four coordinators from Egypt, Morocco, Azerbaijan, and Russia, to explore the oncologists’ demographics, medical training, and background information of responding physicians, in addition to their knowledge and attitudes towards GCSF use in oncology (Supplementary Appendix 1). The link to the survey conducted via Google forms was distributed by the coordinators to oncologists (medical, surgical, radiation, or clinical oncologists) in their local communities and via social media through ESMO and ESO pages. A consent statement was added to be accepted by the participants before proceeding to the questionnaire. Ethical approval for this study was not required.

### Study objectives

The objective of the survey was to describe physician’s knowledge and their practice regarding the daily use of GCSF among cancer patients, including the pandemic context of COVID-19.

### Statistical analysis

Descriptive analyses were conducted on physician’s knowledge and practical attitudes towards GCSF use in oncology. Chi-square test was used to compare the categorical data between groups, and logistic regression was used to find out the possible associations between the prescription patterns. All *p* values below 0.05 were considered statistically significant. The raw data was exported to Microsoft Excel, and the analyses were conducted using IBM SPSS statistics version 20.

## Results

### Demographics

A total of 151 individuals from 4 world regions have completed the survey between 13 July 2021 and 17 September 2021. Table 1 shows the demographic characteristics of the responders. 58.9% of the responses came from the Middle East and North Africa (MENA); Asia, Europe, and Russia shared smaller proportions (9.9, 11.3, and 15.2%) (Table 1).

**Table 1:** Demographic characteristics of the survey responders

Characteristic	*n* (%)
Practice region	
Middle East and North Africa	89 (58.9)
Russia	23 (15.2)
Europe	17 (11.3)
Asia	15 (9.9)
South America	4 (2.6)
South Africa	2 (1.3)
Missing	1 (0.7)
Specialty	
Clinical oncology	66 (43.7)
Medical oncology	67 (44.4)
Radiotherapy	11 (7.3)
Pediatric oncology	3 (2.0)
Onco-hematology	2 (1.3)
Oncology clinical pharmacist	1 (0.7)
Oncology nurse	1 (0.7)
Practice setting	
Academic university hospital	76 (50.3)
Public healthcare	59 (39.1)
Private healthcare	13 (8.6)
Military hospital	1 (0.7)
Missing	2 (1.3)

Half of the participants (50.3%) were practicing in academic hospitals, and the percentage of academic hospital employees was higher among MENA representatives compared with the other regions (*p* < 0.001). Private care workers made up a minority of 8.6%, with 6 (46.2%) of them coming from MENA, 5 (38.5%) from Asia, and 2 (15.4%) from South America. Considering the specialty, clinical and medical oncology accounted for equal numbers (66 and 67 responders, respectively), while radiotherapy gained 7.3% (including 6 radiotherapists from Russia, 3 from Asia, and 2 from MENA).

### Availability

All the responders indicated that G-CSF preparations are available in their daily practice. For 113 (74.8%) participants, the health insurance covers the G-CSF use completely; 23 (15.2%) reported a partial insurance coverage, 13 (8.6%)—no coverage at all, while 2 (1.3%) were not sure about this point. No coverage was reported specifically by some responders from Asia (9 persons), MENA (3 persons), and Europe (1 person), practicing both in an academic and non-academic setting. Among the 136 responders who have G-CSF available within the insurance coverage, 77.9% can prescribe the drug with no limitation, 20.6% limited the prescription to the inpatient setting, and 1.5% can prescribe it in the outpatient setting only.

### Prescribing habits

The block of questions on the prescribing habits included the routine usage of G-CSF prophylaxis, the management of low, intermediate and high-risk chemotherapy-induced afebrile neutropenia, and the systematical risk factor assessment in patients receiving chemotherapy with FN risk of 10–20% (intermediate-risk group).

#### Low-risk group

**Fig. 1:**
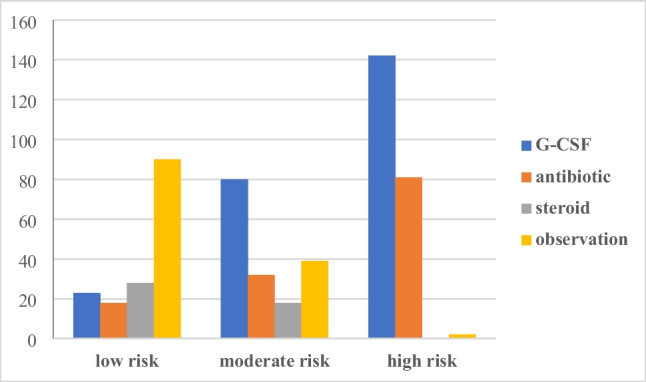
Treatment choices for afebrile neutropenia in the overall responding oncologists

The most common practice in the management of low-risk afebrile cases was observation (57.6% of the responders), with glucocorticosteroid monotherapy prescription ranking second (15.2%) and G-CSF monotherapy was the third (Fig. 1). The corticosteroid prescription rate was high among the Russian representatives, reaching 65.2% (*p* < 0.001 vs. the other regions). None of the responders practicing in Europe, one specialist practicing in Asia, and 12.4% of specialists from MENA chose steroid in low-risk cases.

This bar chart shows the treatment options in the three risk categories: in low-risk group, observation was the commonest strategy, on the contrary to the moderate and high-risk groups where G-CSF was top ranked. Antibiotic was a valid option independent on the risk, while steroid was used in low- and moderate-risk groups.

#### Intermediate-risk group

**Fig. 2:**
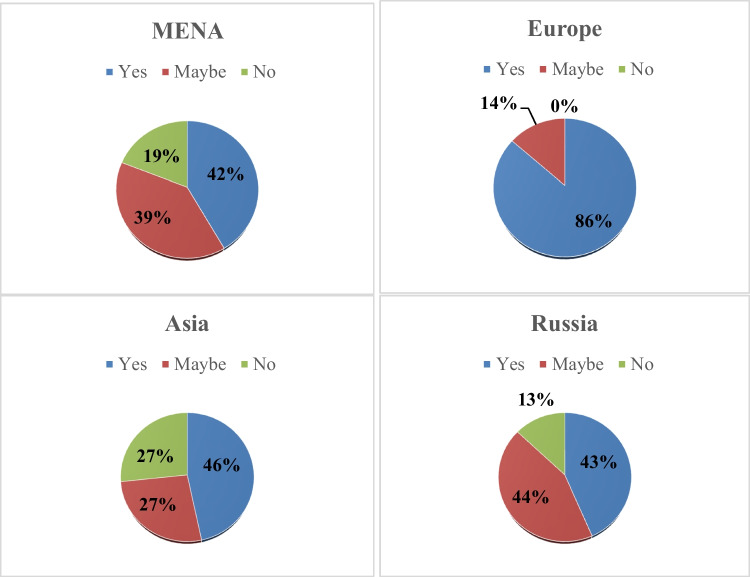
Febrile neutropenia (FN) additional risk factor assessment in different regions

a ***Assessment of additional risk factors in the intermediate-risk group***
  The routine practice of risk factor assessment was indicated as “yes,” “no,” or “maybe.” Among the overall responders, 73 (48.3%) voted for “yes,” 53 (35.1%) voted for “maybe,” and 25 (16.6%) for “no.” The geographical distribution was the only significant factor affecting the routine clinical practice of risk factor assessment; it was significantly common among the specialists settling in Europe—82.4% votes for “yes”—compared with the other regions (*p* < 0.001) which showed the rates ranging from 41.6 to 46.7% for “yes.” Also, 17.6% of the respondents from Europe voted for “maybe,” while this answer was more common in responses from the other regions (Fig. 2). The practice setting (academic vs. non-academic hospitals), *p* = 0.806, the specialty (clinical vs. medical oncology), *p* = 0.935, or the insurance coverage of G-CSF prescription did not affect the rate of the risk factors assessment.
  This figure describes the habits of oncologists in different regions of the world regarding assessing patient and disease risk factors in patients resenting with afebrile neutropenia receiving chemotherapy protocol that carries the risk of 10–20% of developing infection.
b ***Treatment of the intermediate-risk group***
  Regarding the treatment habits in the moderate-risk chemotherapy-induced neutropenia, G-CSF monotherapy presented the most common strategy (43%) followed by observation (23.8%) and prophylactic antibiotics without G-CSF (17.2%) (Fig. 2).
  The use G-CSF in moderate-risk group was more common in non-academic hospitals versus academic institutes (59.7% vs. 40.3%, respectively, *p* = 0.009) in addition to geographical disparities where the MENA showed least frequent rate of GSCF use (39.3%) compared to Russia (95.7%), while the health insurance issues showed no impact. Most of the specialists using G-CSF for moderate-risk cases chose not to prescribe antibiotics (92.4%) or steroids (89.9%) in this group. Only 5 (3.3%) responders combined G-CSF and antibiotics in the moderate-risk group: 3 radiotherapists from Russia, 1 medical oncologist from Europe, and 1 clinical oncologist from the MENA region. The usage of G-CSF in the afebrile neutropenia treatment also was not statistically associated with the risk factor assessment rate.
  The steroid prescription rate was highest in Russia; nine specialists (39.1%). Among the other regions, nine (9.9%) physicians from the MENA chose the steroid. Expectedly, those prescribing steroids in low-risk patients were more likely to prescribe these drugs in the moderate-risk group (OR 8.53, 95% CI 2.96–24.6, *p* < 0.001).

#### High-risk group and primary prophylaxis

a ***Treatment***
  Nearly half (48.3%) of the responders combined G-CSF and prophylactic antibiotics in high-risk afebrile neutropenia. The prescription of G-CSF in high-risk afebrile patients was less frequent for the specialists coming from Europe (76.5%) as compared with the other regions (*p* = 0.015): 86.7% for Asia, 95.5% for MENA, and 100% for Russia. The antibiotic use was the most frequent in Russia—78.3% compared with 48.8% in the other regions (*p* = 0.012) (the antibiotic prescription rate ranged from 35.3 in Europe to 66.7% in Asia). There was also a tendency to a lower rate of antibiotic prescription for high-risk afebrile neutropenia in academic (46.1%) versus non-academic (60.3%) setting, though it did not reach the statistical significance (*p* = 0.082). Where G-CSF is available for the inpatients only, 78.6% of oncologists chose antibiotics compared to 49.1% where there is no limitation to GCSF (*p* = 0.003). There was a statistically significant association between the prescription of antibiotics in the high-risk and the low-risk afebrile neutropenic patients (OR 8.37, 95% CI 1.85–37.83, *p* = 0.006).
b ***Primary prophylaxis***

Around (80%) of the responders chose G-CSF as routine for primary prophylaxis in high-risk patients: 105 (69.5%) voted for “yes” without specifying the percentage of cases with G-CSF prescribed, 11 (7.3%) voted for “yes” mentioning the proportion of more than 50% of cases, and 4 (2.6%) voted for “yes,” but in the amount of less than 50% of cases. Responders who used to assess additional risk factors in their patients (as for “yes” votes) were more likely to use G-CSF prophylaxis routinely (OR 5.08, 95% CI 2.14–12.05, *p* < 0.001). Routine G-CSF prophylaxis usage was also inversely related to the prescription of antibiotics in the univariate analysis (OR 0.41, 95% CI 0.21–0.79, *p* = 0.008), but not in the multivariate analysis.

### The impact of COVID-19 on the rate of GCSF prescription

The current COVID-19 pandemic influenced the rate of prescription of GCSF in the routine practice for 70 (46.4%) responders who increased the G-CSF usage. This practice change was neither associated with the habits concerning risk factor assessment nor with health insurance peculiarities. The increased G-CSF prescription due to the pandemic was more frequent in the MENA representatives (OR 0.75, 95% CI 1.53–6.02, *p* = 0.001) and tended to be positively associated with the reported G-CSF usage in high-risk afebrile neutropenia (OR 4.75, 95% CI 0.94–23.95, *p* = 0.059), but not to any other choices in the field of neutropenia treatment.

## Discussion

All cancer patients may experience at least one episode of neutropenia during the course of the disease [1]. This international collaborative research work highlights the uncertainties in dealing with afebrile neutropenia and emphasizes the need to align the practice with the current guidelines.

The ASCO guidelines recommend “Primary prophylaxis with a CSF starting in the first cycle and continuing through subsequent cycles of chemotherapy is recommended in patients who have an approximately 20% or higher risk for febrile neutropenia based on the patient-, disease-, and treatment-related factors. Secondary prophylaxis with CSFs is recommended for patients who experienced a neutropenic complication from a previous cycle of chemotherapy. CSFs should not be routinely used for patients with neutropenia who are afebrile” [8].

The main focus of this survey was to evaluate the current practice of oncologists worldwide regarding the approach to chemotherapy-induced neutropenia with concerns about the administration of granulocyte colony-stimulating factors (G-CSF) and the impact of the current situation of the COVID-19 pandemic. The responses were quite balanced, an equal number of representatives from academic and non-academic institutions and a full spectrum of physicians treating cancer including medical and clinical oncologists with fewer radiotherapists.

The probability to develop infection secondary to neutropenia depends on the chemotherapy protocol, patient, and disease risk factors; therefore categorize afebrile neutropenic patients into low, intermediate, or high risk. the EORTC stratifies a list of the most common regimens into low, intermediate, and high-risk groups [5].

An intermediate-risk chemotherapy protocol carries a 10–20% probability of neutropenic fever [6, 17], but patient and disease-related factors can upgrade the risk from intermediate to high [5, 18] and thus determining the patient need for GCSF [6]. Patient-related factors include age > 65, poor performance status, comorbidities like cardiac or hepatic disease, recent surgery and/or open wounds, and HIV infection [19]. The disease-related factors are the advanced stage of disease and the previous episode of febrile neutropenia [5] that necessitates secondary prophylaxis [8]. Half of the participants never or unusually assess patients and disease risk factor, and this was confined to responses from Russia and MENA, in contrast to the majority of responders from Europe who routinely assess the patients/disease risk factors. This geographical disparity highlights the need to analyze the underlying obstacles that hinder guideline-based practice like workload or lack of proper knowledge. Risk assessment practice was independent of academic vs. non-academic or medical vs. clinical oncology.

### GCSF prophylaxis for patients with afebrile neutropenia in different risk groups

## The low-risk group

That fits when chemotherapy protocol has a 10% risk to develop sepsis. Observation topped the practice, and steroids came as the second option followed by GCSF. Responses from Russia favored the steroid both in low and intermediate risk neutropenia. There was no evidence in the literature that supports this practice; steroids increases the circulating polymorph-neutrophil by enhancing its migration from the bone marrow and reducing apoptosis [20], but dexamethasone led to earlier and more severe neutropenia when administered as an anti-emetic in bladder cancer patients receiving dose-dense MVAC compared to placebo [21]. Retrospective data showed that 11% of low-risk patients received G-SCF [14], while NCCN, ASCO, and EORTC exclude this group of patients from GCSF prophylaxis [18] [6] [5] and qualify observation as level 1 evidence for practice.

## The intermediate-risk group

G-CSF monotherapy represented the most frequent practice, while observation is the gold standard in patients with no risk factors, and secondary prophylaxis using G-CSF is recommended in patients with 1 or more risk factors [18].The Geriatric Society of Oncology recommends GCSF to senior patients receiving chemotherapy [22]. Lyman et al. provided a prediction tool for complications in patients with neutropenia [23]. The NCCN left the discussion open between the patient and doctor to evaluate the risk–benefit ratio of GCSF use concerning the likelihood of developing FN, the potential consequences of a neutropenia event, and the implications of reduced chemotherapy dose delivery [18], but the ASCO guidelines advise against G-CSFs in patients with neutropenia who are afebrile [8].

The unjustified use of the G-CSF in the intermediate-risk group shown in the prescribing habits of a random sample of oncologists worldwide echoes the patients’ management in routine clinics. A retrospective analysis of patients receiving intermediate-risk chemotherapy protocols showed that secondary prophylaxis with G-CSF was independent of the disease status, metastatic vs. early or solid vs. hematological malignancies [14]. Physician factor shapes the appropriate use of G-CSF [15], and the analysis of the received responses showed that physicians from the academic background were more conservative towards G-CSF prescription than those from the non-academic institutes, in addition to the geographical disparity as doctors practicing in Russia topped the rate of G-CSF prescription, but no difference based on the background of either medical or clinical oncologists. The overuse of G-CSF burdens both the healthcare systems and patients. In the Middle East and Russia—from where most of the responses came—the cost of the course of the GCSF ranges between 30 and 100$ depends if using the generic or bio-similar, and there is an existing debate about its cost-effectiveness [24, 25]. Patients may experience joint and musculoskeletal pain as a reaction to G-CSF administration [26].

## The high-risk group

The combination of G-CSF with antibiotics was the most common practice in patients presenting with neutropenia who are at risk > 20% to develop complications, with geographical preference to Russia and non-academic background. G-SCF in high-risk chemotherapy-induced neutropenia is validated [5, 7] [11], but the combination with antibiotic is debatable. The ASCO and IDSA Clinical Practice Guideline Update recommend in favor of antibiotic prophylaxis using a fluoroquinolone in patients who are at high risk for FN or profound, protracted neutropenia especially in hematological malignancies, but not routinely in solid tumors and when G-CSF prophylaxis effectively reduces the depth and duration of neutropenia [27]. The NCCN [18] and the EORCT [5] recommend GCSF in those patients to reduce the need for antibiotics. Local guidelines should guide the clinical decision due to the absence of solid recommendations at the guideline level.

Primary prophylaxis with GCSF reduces the hospitalization rate and the overall cost of treatment [28] which the majority of physicians favored, but the association between GSCF primary prophylaxis and less antibiotic prescription needs validation. Responses from Europe favored both the risk assessment practice and primary prophylaxis.

### The impact of the COVID-19 on the current practice

Nearly half of the responses detected increase in the G-CSF prescription in response to the pandemic, but we cannot determine if this affected the primary or secondary prophylaxis. The updated ASCO guidelines lowered the cut-off from 20 to 10% to protect more patients in response to the current situation [6]. The NCCN panel extended the use of GCSF into intermediate-risk and low-risk groups where there are comorbidities that could affect the bone marrow [16]. The updated International Society of Geriatric Oncology (SIOG) COVID-19 Working Group [22] recommends the use of GCSF in all advanced age cancer patients receiving chemotherapy as they are more venerable to catch the infection as a result to higher risk of myelosuppression. The G-CSF-associated inflammatory cytokines can augment the interleukin 6 release in patients with COVID infection that negatively impacts their prognosis [29].

### Limitation

One of the limitations we faced, having no responders from the USA meant that we lost a bulk of the practicing oncologists. Also, we did not address the severity of neutropenia as a guiding factor while deciding on the GCSF use. The use of bio-similar in different regions is very interesting regarding the cost-effectiveness and availability, but we deemed to focus on the prescribing habit adjustment.

## Conclusion

Afebrile chemotherapy-induced neutropenia management varies among oncologists based on the geographical and academic backgrounds. The responses revealed underestimation to the importance of risk assessment in the intermediate risk chemotherapy-induced neutropenia and subsequent over prescription of G-CSF in the same group which is inconsistent with the guideline recommendations. This mal-practice was evident in responses from the Middle East and Russia where there are inherent economic challenges to provide cancer treatments. This highlights the need to analyze the underlying obstacles that hinder guideline-based practice like workload or lack of the proper knowledge. The current situation of the COVID-19 pandemic shifts the practice towards generous prophylaxis on both the guideline and clinical practice levels.
